# 2386 Associations of transthoracic echocardiographic features with cardioembolic stroke among patients without atrial fibrillation

**DOI:** 10.1017/cts.2018.47

**Published:** 2018-11-21

**Authors:** Michelle C. Johansen, Nicole L. Williams, Rebecca F. Gottesman

**Affiliations:** Johns Hopkins University School of Medicine

## Abstract

OBJECTIVES/SPECIFIC AIMS: To identify cardiac structural and function parameters, obtained on usual stroke-care TTE evaluation, associated with cardioembolic stroke (CE) in patients without AF. Hypothesis—left atrial (LA) size and valve dysfunction will be strongly associated with incident CE. METHODS/STUDY POPULATION: Inclusion criteria: July 1, 2013 to July 1, 2015 admission with imaging-confirmed ischemic stroke, no AF, TTE within 1st 7 days. TTE structure/function parameters were recorded. Stroke subtype (CE vs. other) defined using TOAST criteria, blinded to TTE. New AF definition: AF on ECG, telemetry or event monitor. CE/New AF outcome of interest in separate multivariable logistic regression models testing associations with TTE parameters (adjusting for demographics/vascular risk factors). RESULTS/ANTICIPATED RESULTS: Participants (n=332) were ~60 years hypertensive black males with moderate NIHSS and normal ejection fraction. In adjusted models, odds of CE increased with increasing LA systolic diameter (per 0.1 cm), mitral E point velocity(cm/s), mitral valve dysfunction, wall motion abnormality. New AF also associated with increasing LA systolic diameter. DISCUSSION/SIGNIFICANCE OF IMPACT: These findings may suggest cardiac structural changes independent of AF that are on the CE causal pathway. Understanding the relationship between such TTE parameters and stroke subtype would impact clinical practice, as such TTE data is underutilized when considering stroke mechanism and management.

